# A pilot program of HIV pre-exposure prophylaxis in Thai youth

**DOI:** 10.1371/journal.pone.0298914

**Published:** 2024-02-22

**Authors:** Supattra Rungmaitree, Peerawong Werarak, Wadchara Pumpradit, Wanatpreeya Phongsamart, Keswadee Lapphra, Orasri Wittawatmongkol, Yuitiang Durier, Alan Maleesatharn, Beena Kuttiparambil, Tim R. Cressey, Risa M. Hoffman, Kulkanya Chokephaibulkit

**Affiliations:** 1 Department of Pediatrics, Faculty of Medicine Siriraj Hospital, Mahidol University, Bangkok, Thailand; 2 Department of preventive and social medicine, Faculty of Medicine Siriraj Hospital, Mahidol University, Bangkok, Thailand; 3 Bangkok Health Hub Medical Centre, Bangkok, Thailand; 4 UNICEF Thailand, Bangkok, Thailand; 5 PHPT/IRD UMI 174, Faculty of Associated Medical Sciences, Chiang Mai University, Chiang Mai, Thailand; 6 Division of Infectious Diseases, Department of Medicine, David Geffen School of Medicine at the University of California, Los Angeles, California, United States of America; 7 Siriraj Institute of Clinical Research, Faculty of Medicine Siriraj Hospital, Mahidol University, Bangkok, Thailand; University of Toronto, CANADA

## Abstract

**Introduction:**

There are gaps in knowledge and experience of antiretroviral pre-exposure prophylaxis (PrEP) delivery in adolescents.

**Methods:**

This pilot study enrolled Thai adolescents 14–20 year-old without HIV who reported risk behaviour. All participants were offered daily tenofovir/emtricitabine (TDF-FTC) and followed for 24 weeks. HIV testing, renal function, bone density scan, and sexually transmitted infection (STI) testing including syphilis serology and urine molecular testing for gonorrhoea and *C*. *trachomatis* were performed at baseline and weeks 12 and 24. Adherence was evaluated through intracellular tenofovir diphosphate (TFV-DP) levels in dried blood spots.

**Results:**

Of the 61 enrolled adolescents, median age 18.1 (IQR: 14.8–20.9) years, 46 (75.4%) were males and 36 (59%) were MSM. Retention to week 24 was 80.3%. One third (36%) had TFV-DP levels consistent with taking ≥6 pills/week at week 12 and 29% at week 24. The factors associated with taking ≥6 pills/week were being MSM (adjusted odds ratio [aOR]: 53.2, 95% CI: 1.6–1811; *p* = 0.027), presence of STI at baseline (aOR: 9.4, 95% CI: 1.5–58.5; *p* = 0.016), and self-report of decreased condom use while taking PrEP (aOR: 8.7, 95% CI: 1.4–56.6; *p* = 0.023). 31% had an STI at baseline and this declined to 18% at week 24. No renal or bone toxicity was observed and there were no HIV seroconversions.

**Conclusions:**

Daily oral PrEP with FTC-TDF in high-risk Thai adolescents is feasible, accepted, well-tolerated, and had no increased risk compensation; however, low adherence was a major challenge. Adolescent-specific PrEP strategies including long-acting modalities are needed for successful HIV prevention.

## Introduction

Human immunodeficiency virus (HIV) is a major public health issue, with over 38.4 million people living with HIV globally and 1.5 million people newly infected in 2021 [1, 2]. Adolescents and young adults (15–24 years) including young men who have sex with men (YMSM) bear a disproportionate burden of HIV and account for over a third of new HIV infection globally, with over 160,000 new infections among 10–19 years old within 2021 alone [3]. Similarly, in Thailand the national AIDS prevention and alleviation committee reported that 50% of HIV incident infections are among young people aged 15–24 years and the majority of these were YMSM [4]. Adolescents have distinct socio-behavioural patterns that increase their risk of HIV infection, including high rates of intravenous drug use [5], frequent high-risk sex, income generation through sex work, or a combination of these factors [5–9]. Efforts to prevent HIV in adolescents are needed to curb the global HIV epidemic.

Pre-exposure prophylaxis (PrEP) with daily combined tenofovir disoproxil fumarate and emtricitabine (TDF/FTC), is one of the highly effective approaches for the HIV prevention in adult population at risk of acquiring HIV with efficacy ranging from 62–75% in heterosexual couples and 44–86% in MSM [10–14]. In these studies, optimal adherence further increased the efficacy of PrEP to 92% [14]. In 2012, the TDF/FTC was approved by the Food and Drug Administration (FDA) for use as PrEP in adult [15]. Since then, recognition has been increasing that adolescents at risk for acquiring HIV can benefit from PrEP. In 2018, the FDA expanded the indication for PrEP to include adolescents weighing at least 35 kg. who are at risk for acquiring HIV [16].

In 2014, PrEP has been included in the Thai National Guidelines on HIV/AIDS Treatment and Prevention and recommended for adults who are at high-risk individuals, specifically MSM, available for self-pay and through demonstration projects. Then PrEP become available free of charge under the national health universal coverage since October 2019. Moreover, the medical council recommended that adolescents aged 13 to 18 years can get HIV testing and treatment without parental consent since 2015 [17, 18]. As of 2022, a PrEP coverage under a government-supported program revealed that of 3,134 HIV-negative MSM and TGW offered PrEP, 1,896 (60.5%) accepted to take PrEP and 1,174 (61.9%) retained on PrEP. However, only 4.8% of all PrEP prescriptions were for adolescents aged 15–19 despite their at extremely high-risk of HIV infection [19]. Despite a growing body of published experience with PrEP in adults, a minority of previous research has included youth under age 20, particularly from Asian settings. Previously, PrEP studies from the US, Adolescent Trials Network (ATN) 110 and ATN 113 indicated that youth were likely interested and appeared benefit from PrEP; however, adherence is a potential barrier to effective use of PrEP in this population [20, 21]. To help inform PrEP implementation in Thailand, we designed a PrEP demonstration study among adolescents aged 14 to 20 years with the primary goal is to characterize retention in care and adherence to PrEP. We secondarily examined factors associated with PrEP retention and adherence, the safety and tolerability of daily PrEP and change in sexual risk compensation among adolescent PrEP users.

## Methods

We conducted the PrEP service delivery program between January 2019 and September 2019 at 2 centres in Bangkok: The Department of Paediatrics and Department of Preventive and Social Medicine at Siriraj Hospital, Mahidol University, and Bangkok Health Hub, a private clinic that provides comprehensive sexual health services, including gender affirming hormone treatment. Institutional Review Board approval was granted by the Faculty of Medicine Siriraj Hospital Mahidol University with a waiver for parental consent granted (approval no. Si333/2561).

### Recruitment and eligibility

Multiple recruitment methods were employed including online outreach, school and vocational school presentations, network and peer-to-peer referrals, and Mor-Lam concert outreach (North-eastern Thai folk performance attractive to MSM community). For the online outreach, we developed a Thai website for young people (https://ym2m.lovecarestation.com) to provide education about reproductive health, sex, and offered online counselling. All participants completed the registration form voluntarily before initiating online counselling. Online counsellors conducted risk assessment, provided HIV and STI prevention knowledge, introduced HIV testing and PrEP services, and linkage to HIV testing. For network referral, the primary- and secondary-care hospitals that routinely refer HIV-infected patients to our center are informed about this study. The adolescents who deserve PrEP were referred to our center if they are interested in the study. Eligible participants were adolescents without HIV, aged 14 to 20 years, with HIV transmission risk behaviour within the past 6 months, defined as follows: partner with HIV either recently started or not taking antiretroviral therapy (ART); inconsistent condom use with partner HIV status unknown; being MSM; recent STI; and/or a sex worker. Adolescents were ineligible if they had creatinine clearance (CrCl) <60 mL/min calculated based on the Cockroft-Gault equation, had a history of unexplained bone fracture, known allergy or sensitivity to TDF/FTC, or unwilling to participate.

### Study procedures

After informed consent (for all participants aged 18 years or older) or assent (for participants younger than 18 years), participants were scheduled to attend clinical visits at week 0, 4, 12, and 24. The participants received approximately 30 U$ for each visit to for travel expenses and as compensation for time spent. All participants received a comprehensive package for HIV prevention services included sexual risk-reduction counselling with provision of condom, lubricants, screening, and treatment for STIs, and all were dispended a 30-day of TDF/FTC tablets at the first visit, then a 90-day of TDF/FTC tablets were supplied in the subsequent visits. At each study visit, adherence, and sexual risk behavioral survey were captured by participant responses to self-administered paper questionnaires. The questionnaires included specific questions such as “number of pills taken per week since last visit”, “having any side effects from PrEP”, “numbers of sex partners in the past month”, “consistent condom use during all episode of sexual intercourse” and “any change in condom used while taking PrEP”.

At study visit, laboratory data were collected. Laboratory evaluations included hepatitis-B surface antigen (HBsAg) and antibody tests (HBsAb) (only baseline), HIV antibody using 4^th^ generation enzyme-linked immunosorbent assay (ELISA), creatinine, urinalysis, and urine pregnancy tests (for female participants). Whole body dual-energy X-ray absorptiometry (DXA) scans and STIs screening was performed at week 0 and week 24; the latter included: venereal disease research laboratory (VDRL), urine for nucleic-acid amplification testing (NAAT) for gonorrhoea (GC) and *Chlamydia trachomatis* (CT). Intracellular tenofovir diphosphate (TFV-DP) concentrations were measured at week 12 and 24 through dried blood spot (DBS) samples collected onto Whatman Protein Saver 903 cards and stored at -70°C until analysis. TFV-DP was analysed using liquid chromatography mass spectrometry (LC-MS/MS).

Participants who were positive for HBsAg and negative for HBsAb at baseline were also tested for liver function tests including aspartate aminotransferase (AST) and alanine aminotransferase (ALT), and the individuals were excluded from the study if they had elevations AST or ALT more than 3-fold the upper limit. Participants found to be non-immune to hepatitis B at baseline were offered hepatitis B vaccination starting at their enrolment visit or at any time thereafter during study participation. Enrolled individuals with a positive HBsAg were monitored for hepatitis flares through the study and after stopping PrEP.

### Outcomes

The primary outcomes included retention in care and adherence to PrEP. Retention was defined by attendance to the study visit until the end of the study visit at week 24. Adherence was measured with intracellular TFV-DP concentration. Based on a pharmacokinetic and pharmacodynamic (PK/PD) data by measuring mucosal tissue concentrations of TFV-DP indicated that colorectal TFV-DP concentration was 10 times higher than that in the lower female genital tract. A minimal adherence to ≥4 doses/week was required to protect colorectal tissue, while minimum adherence to 6 doses/week was required to protect lower female genital tract tissue [22]. Sufficient adherence was defined as TFV-DP ≥700 fmol/punch equivalent to ≥4 pills/week for male participants, and TFV-DP ≥1050 fmol/punch equivalent to ≥6 pills/week for female participants [23].

The secondary outcomes included factors associated with retention and adherence to PrEP, safety and tolerability of daily PrEP, and changes in behavioural risk while taking PrEP. Safety and tolerability were monitored with CrCl and BMD, and all other types of adverse events were self-reported in the questionnaires. Sexual risk compensation was measured as the proportion of participants with STIs at baseline and week 24 visits.

### Statistical analysis

Baseline demographics, retention, adherence, clinical adverse events (AEs), STIs, and measured level of drug exposure were summarized using frequencies, means, medians, standard deviations, and ranges, as appropriate. The BMD Z-scores were generated by DXA software using Thai children and adolescent norms [24]. Univariate and multivariate logistic regression analyses were performed to identify factors associated with PrEP retention and adherence. Factors associated with sufficient and non-sufficient adherence to PrEP, using sex-specific cut-offs for TFV-DP levels as above were analyzed at week 12 and 24. Covariate included demographic data and self-report information from questionnaires. Covariate with p-values <0.05 on univariate analyses was entered into multivariate regression analysis using adjusted odd ratios (aOR) with statistical significance defined as p-values <0.05. All statistical analyses were performed using STATA 15.1 (Stata Corp, Texas, USA).

## Results

### Participant characteristics

**Fig 1:**
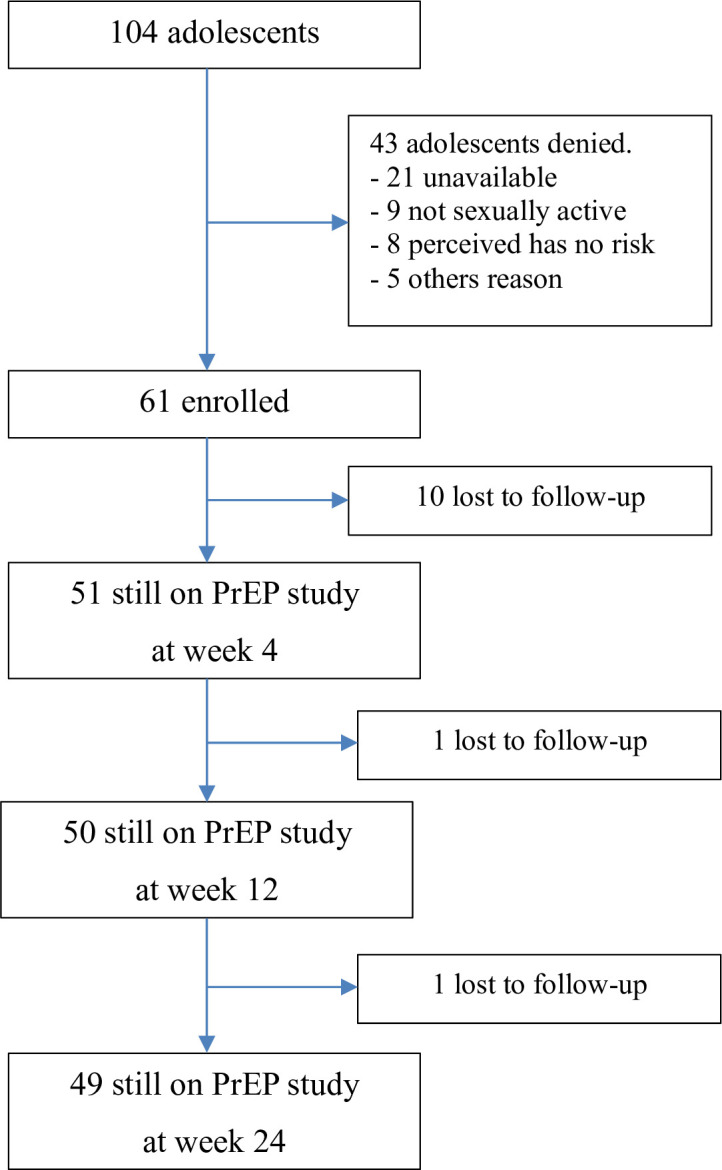
Study profile.

Between January 2019 and September 2019, 104 adolescents were approached and 61 (58.7%) consented to screening. Of 43 adolescents who declined, the most common reasons were being unavailable for all study visits and procedures (n = 21), no current sexual partner (n = 9), and perceived no risk of HIV (n = 8) (Fig 1). Of the 61 participants enrolled, the median age was 18.1 years (IQR: 14.8–20.9), 46 (75.4%) were males and 36 of these individuals (59%) self-reported as MSM. Two participants (3.3%) had chronic hepatitis B infection, 38 (62.3%) reported previous testing for HIV, and 8 (13.1%) reported prior PrEP use. Most participants (88.5%) reported 1–3 sexual partners in the past month (Table 1).

**Table 1:** Participant demographics and characteristics.

Characteristics	(N = 61)
Biological sex, n (%)	
Male	46 (75.4)
Female	15 (24.6)
Age at enrolment, median (IQR), year	18.1 (14.8–20.9)
Hepatitis B status, n (%)	
Immune to hepatitis B	13 (21.3)
Non-immune to hepatitis B	46 (75.4)
Chronic hepatitis B infection	2 (3.3)
Risk to take PrEP, n (%)	
MSM	36 (59.0)
Serodiscordant couple	6 (9.8)
FSW	1 (1.6)
Risky sexual behaviour	18 (29.5)
Prior HIV testing, n (%)	
Yes	38 (62.3)
No	23 (37.7)
Prior PrEP taking, n (%)	
Yes	8 (13.1)
No	53 (86.9)
Number of sex partners in the past month, median (IQR), n (%)	1 (0–10)
None	3 (4.9)
1–3	54 (88.5)
4–6	3 (4.9)
≥ 7	1 (1.6)

Abbreviations: PrEP, Pre-exposure phophylaxis; MSM, Men who have sex with men; FSW, female sex worker

### Participants retention

Retention in the PrEP program at week 24 was 49/61 (80.3%). Twelve participants (19.7%) were lost to follow-up after the week 4 visit (n = 10) (Fig 1). For those that remained on the study after week 4 (n = 51), visit retention was very high with 96.1% of all expected visits (Table 2). In logistic regression, the only factor that associated with retention at week 24 was being MSM (odds ratio [OR]: 5.1; 95% confidential interval [CI]: 1.2–20.8, *p* = 0.023) (S1 Table).

**Table 2:** Participants retention and sexual risk compensation evaluation.

Variable	Baseline N = 61	Week 4 N = 51	Week 12 N = 50	Week 24 N = 49
Participant retention, n (%)	61 (100)	51 (83.6)	50 (81.9)	49 (80.3)
Negative for anti-HIV, n (%)	61 (100)	51 (100)	50 (100)	49 (100)
Having STDs, n (%)	19 (31.2)	-	-	9 (18.4)
Syphilis	2 (3.3)	-	-	1 (2.0)^†^
*Chlamydia trachomatis*	10 (16.4)	-	-	7 (14.3)^‡^
*Neisseria gonorrhoea*	7 (11.5)	-	-	1 (2.0)^§^
Negative pregnancy result^¶^, n (%)	15 (100)	11 (100)	11 (100)	11 (100)

^†^New infected cases of syphilis; ^‡^3 re-infected and 4 new cases of *C*. *trachomatis*; and ^§^new infected cases of *N*. *gonorrhoea*. ^¶^For female adolescents only.

### Assessment of PrEP adherence by self-report and TFV-DP concentrations

**Fig 2:**
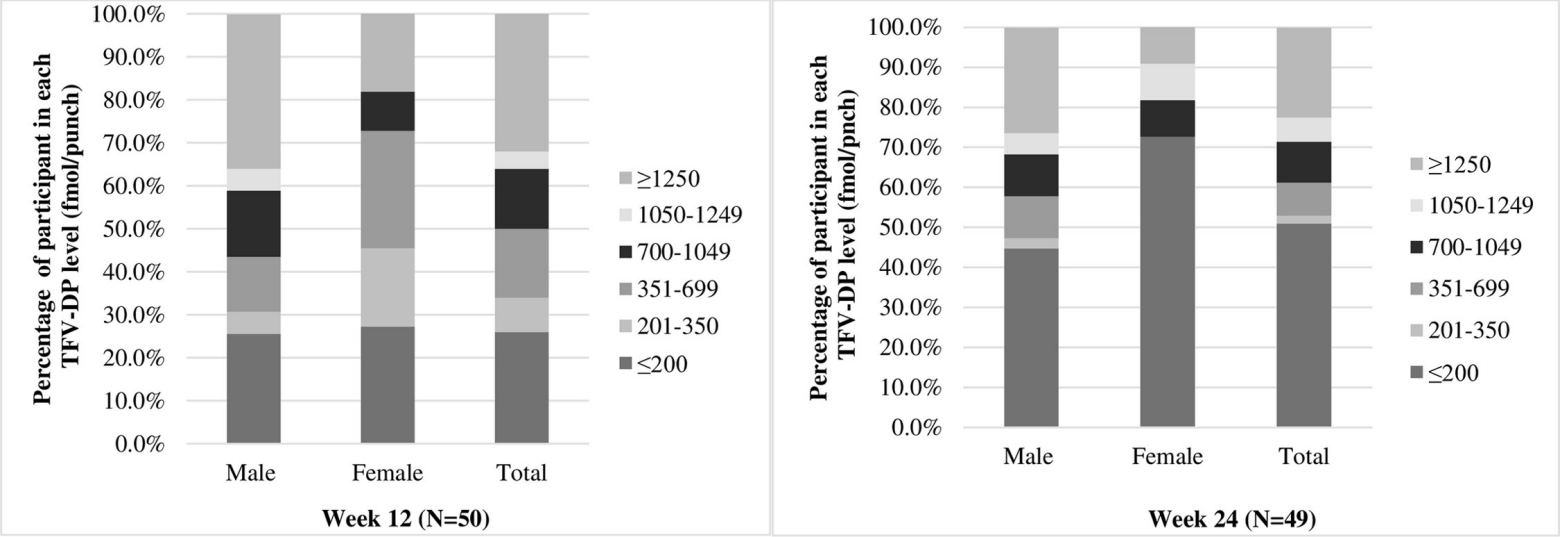
Adherence to pre-exposure prophylaxis treatment in participants determined by TFV-DP concentration at week 12 and 24. TFV-DP categories were based on ≥ 700 fmol/punch for ≥ 4 doses/week, ≥ 1050 fmol/punch for ≥ 6 doses/week, and ≥ 1250 fmol/punch for 7 doses/week.

Ninety-five percent and 78.9% of male participants self-reported taking PrEP ≥4 pills/week at week 12 and week 24, respectively, and 63.6% and 36.4% of female participants reported taking PrEP ≥6 pills/week, at week 12 and week 24, respectively (S2 Table). Quantitative analysis of TFV-DP levels indicated that 36.0% of participants had TFV-DP levels ≥1050 fmol/punch (equal to ≥6 pills/week) at week 12, and 28.6% at week 24. In male participants, 56.4% and 42.1% had TFV-DP level ≥700 fmol/punch (equal to ≥4 pills/week) at week 12 and week 24, respectively. In female participants, 18.2% had TFV-DP levels ≥1050 fmol/punch, equal to ≥6 pills/week, at both week 12 and 24 (Fig 2 and S2 Table).

### Factors associated with PrEP adherence

Factors associated with taking ≥6 pills/week as determined by TFV/DP levels at week 12 were being MSM (adjusted odds ratio [aOR]: 53.2, 95% CI: 1.6–1,811; *p* = 0.027), and presence of STIs at screening (aOR: 85.5, 95% CI: 1.9–3,816; *p* = 0.022) (S3 Table). At week 24, STIs at screening remained significant (aOR: 9.4, 95% CI: 1.5–58.5; *p* = 0.016) and self-report of decreased condom use while taking PrEP was additionally associated with taking ≥6 pills/week (aOR: 8.7, 95% CI: 1.4–56.6; *p* = 0.023) (Table 3). For males, factors associated with taking ≥4 pills/week at week 12 was having condomless sexual intercourse in the past 3 months (aOR: 6.0, 95% CI 1.0–34.9; *p* = 0.045); and at week 24, receiving PrEP from the private clinic (OR 7.8, 95% CI 1.4–43.1; *p* = 0.018) (S4 and S5 Tables).

**Table 3:** Factors associated with adherence to pre-exposure prophylaxis treatment among participants retained at week 24.

Variables	Consistent < 6 pills/week (<1050 fmol/ punch) (N = 35)	Consistent ≥ 6 pills/week (≥1050 fmol/punch) (N = 14)	P-value	Multivariate analysis	
				Adjusted OR (95% CI)	P-value
Biological sex, n (%)					
Female	9 (25.7)	2 (14.3)		-	-
Male	26 (74.3)	12 (85.7)	0.393		
Age at enrolment, median (range), years	17.9 (14.9–20.8)	19.5 (15.7–20.9)			
< 18	18 (51.4)	3 (21.4)		-	-
≥ 18	17 (48.6)	11 (78.6)	0.065		
Enrolment clinic, n (%)					
Adult HIV clinic	15 (42.9)	1 (7.1)			
Private sexual health clinic	18 (51.4)	12 (85.7)	**0.036**	6.9 (0.9–51.3)	0.059
Paediatric HIV clinic	2 (5.7)	1 (7.1)	0.209		
Prior HIV testing; n (%)					
Yes	20 (57.1)	12 (85.7)	0.072	-	-
No	15 (42.9)	2 (14.3)			
Risks to take PrEP, n (%)					
Serodiscordant					
Yes	4 (11.4)	2 (14.3)	0.783		
No	31 (88.6)	12 (85.7)		-	-
Inconsistent condom use					
Yes	29 (82.9)	14 (100.0)	0	-	-
No	6 (17.1)	0			
MSM					
Yes	20 (57.1)	12 (85.7)	0.072	-	-
No	15 (42.9)	2 (14.3)			
Having STIs at screening, n (%)					
Yes	4 (11.4)	8 (57.1)	**0.002**	**9.4 (1.5–58.5)**	**0.016**
No	31 (88.6)	6 (42.9)			
Experienced AEs from PrEP, n (%)					
Yes	7 (20.0)	2 (14.3)	0.505	-	-
No	28 (80.0)	12 (85.7)			
Had difficulty taking PrEP*, n (%)					
Yes	16 (45.7)	4 (28.6)	0.275	-	-
No	19 (54.3)	10 (71.4)			
No. of sex partner in the past month, n (%)					
≤ 1	29 (82.9)	7 (50.0)			
≥ 2	6 (17.1)	7 (50.0)	0.024	5.4 (0.9–34.4)	0.073
Decreased condom use while taking PrEP, n (%)					
Yes	7 (20.0)	7 (50.0)	**0.042**	**8.7 (1.4–56.6)**	**0.023**
No	28 (80.0)	7 (50.0)			
Current alcohol use, n (%)					
Yes	30 (85.7)	10 (71.4)	0.252	-	-
No	5 (14.3)	4 (28.6)			
Current smoking, n (%)					
Yes	11 (31.4)	3 (21.4)	0.487	-	-
No	24 (68.6)	11 (78.6)			

*Based on stigma, concern others would see pills, social pressures, or a combination of these factors

### Safety and sexual risk compensation

There were 41 self-reported adverse events (AEs) among 51 participants. The most common AEs were nausea (n = 11; 21.6%), dizziness (n = 11; 21.6%), and appetite loss (n = 4; 7.8%), all of which were mild. None of participants discontinued PrEP due to symptoms and all symptoms were fully resolved without discontinuation (S6 Table). Amongst the participants who had BMD performed at baseline and week 24, BMD Z-scores did not significantly change (*p* = 0.90), and median BMD slightly increased with statistically significant (median +1.8%, *p* = 0.01). CrCl increased between baseline and week 24 (+7.6 mL/min, *p* = 0.04) although this degree of change was not clinically significant (S7 Table). No evidence of risk compensation, the STIs rate among participants at the baseline visit was 31.2%, and the rate was lower at week 24 (18.4%), with the STIs incidence rate of 18.3 per 100 person-years during the study. None of the participants had HIV seroconversion during the study (Table 2).

## Discussion

This study demonstrated that a cohort of youth at high risk for HIV acquisition in Thailand can be recruited, enrolled, and retained in an adolescent PrEP service delivery program. The comprehensive package for prevention provided while participating in the program including counselling, travel compensation, and other reproductive health services, was likely important for retention, and these types of wraparound services have been important in other adolescent PrEP studies as well [25]. While self-reported adherence was relatively high over the course of the study, TFV-DP blood levels indicated actual adherence was approximately half of the levels indicated by self-reported. In this study, we found about half of male participants had sufficient adherence with TFV-DP levels consistent with ≥4 pills/week at week 12 and 24. Similar adherence challenge have been seen in Adolescent Trials Network for HIV/AIDS Interventions (ATN) protocol 110 (ages 18–22 years) and 113 (ages 15–17 years) in the US, and previous Thai YMSM PrEP trial (ages 15–19 years), with protective PrEP levels being observed in 30–50% of adolescents through week 24 [20, 21, 26]. In female participants, we found only 18% had TFV-DP levels indicating adherence to ≥6 pills/week at both 12 and 24 weeks, and similar data have been reported from young African women who received PrEP, in which adequate TDF levels were achieved in only 5–25% of the population [27, 28]. Although PrEP adherence in this pilot program was low, the high retention rates are consistent with other studies [20, 29]. These findings suggest that PrEP treatment that does not require daily adherence such as long-acting injectable agents may be more effective approach for HIV prevention among high-risk adolescents.

We found that the highest rate of discontinuing PrEP program was 16.4% (10/61) at week 4. The most prevalent reason for discontinuing PrEP after interviewing via phone for those who did not attend on subsequent visits was that they were no longer at risk of HIV transmission i.e., abstaining from sexual relations and/or entering a monogamous relationship. This is similar to the previously reported on PrEP use by MSM and TGW [30, 31]. It is also due to gaining knowledge while receiving PrEP. Nonetheless, the perception of personnel HIV risk is dynamic. It is important to help adolescents understand how to accurately assess individuals’ risk, so they can make optimal decisions especially when considering stopping or re-starting PrEP consistent with prevention-effective adherence [32].

We found that being MSM, having STIs at screening, and decreased condom use while on PrEP were associated with better PrEP adherence. Similarly, the ATN 110 study demonstrated that higher TFV-DP concentrations were seen among participants that reported condomless sex with their partner [20]. Data from prevention clinical trials suggests that higher perception of HIV risk is associated with high oral PrEP adherence [33–35]. In contrast, a study among adolescents and young adults with MSM in US did not find risk perception to be associated with PrEP adherence [36]. Regarding low adherence among women, some potential PrEP stigma attitudes were identified during counseling session that they were concerned about others would assume that a PrEP user was HIV infected, or had multiple sex partners. This is in accordance with the previous oral PrEP trials in adolescent girls and young women that experiencing stigma and led to concealment of product use and subsequent lower adherence [37]. We found adolescents who received PrEP at private clinic (Bangkok Health Hub) had better adherence rates than in the academic setting. In our setting, private clinic was available out-of-office hour, have shorter wait times and comprehensive sexual health services, including gender affirming hormone therapy and this may have created an environment with stronger support services that influenced adherence. Furthermore, one qualitative study among YMSM and young transgender women in Thailand indicated that a more adolescent friendly service, i.e., not stigmatized, fast, convenient, and inclusive of other services such as gender-affirming treatment, may improve the adherence and the success of PrEP programs [38]. In this study we provided in person-counselling to improving adherence. We found similar adherence level compared to a previous YMSM PrEP trial in Thailand that used mobile phone application to promote adherence [26]. This is consistent with a recent systematic review of factors moderating adherence that revealed no specific PrEP strategies (i.e. in-person adherence, smartphone applications, and in-person counselling) effectively promote adherence in adolescent and young adult [39]. New development of methods to effectively sustain PrEP adherence in adolescent is needed.

One concern about PrEP as an HIV prevention strategy is the risk compensation due to the relaxation of safe-sex practice among those who feel confident of protection from taking PrEP. This evidence is available from a systematic review in adult MSM that shown an increase in condomless sex among PrEP users, with a significant increase in STIs [40]. In our study, 83.7% (41/49) of participant who retained in the PrEP program reported inconsistent condom used or condomless practice. From self-report, 12.8% (5/39), and 28.9% (11/38) of MSM participants reported decreased condom use while taking PrEP at week 12 and 24, respectively. However, there was no evidence of risk compensation, consistent with previous PrEP studies in adolescents, including ATN studies 110 and 113 [20, 21]. We found the rate of STIs decreased from 31% at baseline to 18% at week 24; however, the overall STI rate was high compared with prior PrEP trials in young adolescents [21], underscoring the importance of STI prevention with specific counselling adapted for adolescents.

We found no evidence of significant PrEP toxicities, such as decreased BMD or reduced renal function. Our data are consistent with safety data from other PrEP trials in adolescents and young adults, which showed no significant bone events and no PrEP interruption occurred due to renal impairment [20, 21, 41]. In our population, PrEP was well tolerated, with no documented discontinuations due to AEs or abnormal laboratory results. However, our sample size was small and not powered to detect smaller changes over the relatively short follow-up period of 24 weeks. Additionally, non-adherence to PrEP was substantial, so overall exposure to TDF/FTC was low, limiting our ability to measure PrEP-related safety outcomes. Future studies should be performed with longer durations of follow-up to evaluate changes in BMD and as well as safety and toxicity. Accurate assessments can only be fully made in the setting of moderate to high adherence, which remain a consistent challenge in adolescent population globally. Additionally, among the 2 participants enrolled in the study with chronic hepatitis B, neither experienced a hepatitis B flare, either during the trial or after drug discontinuation. This result is consistent with previous adult MSM PrEP trials, which have shown the safety of TDF-FTC PrEP in individuals with HBV infection if there is no evidence of substantial transaminase elevation [42].

This study has several limitations. The relatively small number of participants and predominantly cisgender male population who identified as MSM, which limit the generalizability of the findings to other geographic areas or sociodemographic categories. However, young MSM is the predominate at risk population in Thailand to which this study could be applied. The results in females need to be interpreted with caution as there were a small number in our sample. Finally, 24-week follow-up limits our ability to detect change over a longer duration, which is likely to be important for certain outcomes, such as BMD. Finally, outcomes were seen in the setting of low overall PrEP adherence, which is an important limitation of our safety results.

## Conclusions

Our data suggest that PrEP with daily TDF/FTC for adolescents in urban Thailand is feasible, well-accepted, well-tolerated, and is not associated with risk compensation. Low PrEP adherence is a major challenge in this population and strategies such as on-demand PrEP (for those who qualify) or long-acting PrEP agent that would likely reduce the adherence issue in high-risk Thai adolescents. Youth-specific adherence intervention strategies and peer support systems are required for successful HIV and STI prevention, particularly for female adolescents in whom higher adherence is needed to achieve effective drug levels. Adolescents are open to engaging with healthcare services and require quality counselling and comprehensive sexual health services to generate risk awareness and improve health outcomes.

## Supporting information

S1 Table Factors associated with retention in the PrEP program. (DOCX)

S2 Table TFV-DP concentrations among participants by gender and adherence at week 12 and 24. (DOCX)

S3 Table Factors associated with adherence to pre-exposure prophylaxis treatment among participants retained at week 12. (DOCX)

S4 Table Factors associated with adherence to pre-exposure prophylaxis treatment among male participants retained at week 12. (DOCX)

S5 Table Factors associated with adherence to pre-exposure prophylaxis treatment among male participant retained at week 24. (DOCX)

S6 Table Self-reported adverse events in adolescents while taking tenofovir disoproxil fumarate-emtricitabine for pre-exposure prophylaxis. (DOCX)

S7 Table Bone mineral density and kidney function in adolescents taking tenofovir disoproxil fumarate-emtricitabine for pre-exposure prophylaxis. (DOCX)
