# Supplementary material for: A pilot program of HIV pre-exposure prophylaxis in Thai youth
Source: PLoS One. 2024 Feb 22;19(2):e0298914. doi: 10.1371/journal.pone.0298914 (PMC10883585; doi:10.1371/journal.pone.0298914)
Supplement: S1 Table — (DOCX) [file pone.0298914.s001.docx]

**S1 Table.** Factors associated with retention in the PrEP program.

| **Variables** | **Not retained**  **(N = 12)** | **Retained**  **(N = 49)** | **Crude OR**  **(95% CI)** | **P-value** |
| --- | --- | --- | --- | --- |
| Biological sex, n (%) |  |  |  |  |
| Female | 4 (33.3) | 11 (22.4) | 1 |  |
| Male | 8 (66.7) | 38 (77.6) | 1.7 (0.4-6.8) | 0.436 |
| Age at enrolment, median (range), year | 18.3 (14.8-20.5) | 18.1 (14.9-20.9) |  |  |
| < 18 | 6 (50.0) | 21 (42.9) | 1 |  |
| ≥ 18 | 6 (50.0) | 28 (57.1) | 1.3 (0.4-4.7) | 0.656 |
| Enrolment clinic, n (%) |  |  |  |  |
| Adult HIV clinic | 3 (25.0) | 3 (6.1) | 1 |  |
| Private sexual health clinic | 6 (50.0) | 30 (61.2) | 5 (0.8-31.0) | 0.084 |
| Paediatric HIV clinic | 3 (25.0) | 16 (32.7) | 5.3 (0.7-40.2) | 0.104 |
| Risks to take PrEP, n (%) |  |  |  |  |
| Risky sexual behaviour | 7 (58.3) | 11 (22.4) | 1 |  |
| MSM | 4 (33.3) | 32 (65.3) | 5.1 (1.2-20.8) | **0.023** |
| Serodiscordant couple | 1 (8.3) | 5 (10.2) | 3.2 (0.3-33.3) | 0.334 |
| No. of sex partner in the past month, median (range), n (%) | 1 (1-3) | 1 (0-10) |  |  |
| ≤ 1 | 7 (58.3) | 34 (69.4) | 1 |  |
| ≥ 2 | 5 (41.7) | 15 (30.6) | 0.6 (0.2-2.3) | 0.467 |
| Prior HIV testing; n (%), |  |  |  |  |
| Yes | 6 (50.0) | 32 (65.3) | 1.9 (0.5-6.7) | 0.331 |
| No | 6 (50.0) | 17 (34.7) | 1 |  |
| Prior PrEP taking, n (%) |  |  |  |  |
| Yes | 11 (91.7) | 42 (85.7) | 0.5 (0.1-4.9) | 0.589 |
| No | 1 (8.3) | 7 (14.3) | 1 |  |
| Having syphilis, n (%) |  |  |  |  |
| Yes | 2 (16.7) | 0 | - | - |
| No | 10 (83.3) | 49 (100.0) |  |  |
| Having GC infection, n (%) |  |  |  |  |
| Yes | 1 (8.3) | 6 (12.2) | 1.5 (0.2-14.1) | 0.705 |
| No | 11 (91.7) | 43 (87.8) | 1 |  |
| Having CT infection, n (%), |  |  |  |  |
| Yes | 3 (25.0) | 7 (14.3) | 0.5 (0.1-2.3) | 0.375 |
| No | 9 (75.0) | 42 (85.7) | 1 |  |
| Hepatitis B status, n (%) |  |  |  |  |
| Immune | 3 (25.0) | 10 (20.4) | 3.3 (0.2-70.9) | 0.440 |
| Non-immune | 8 (66.7) | 38 (77.6) | 4.7 (0.3-84.2) | 0.288 |
| Chronic hepatitis B | 1 (8.3) | 1 (2.0) | 1 |  |
| Partner HIV status, n (%), |  |  |  |  |
| Unknown | 4 (33.3) | 31 (63.3) | 1 |  |
| Infected | 3 (25.0) | 5 (10.2) | 0.2 (0.1-1.3) | 0.089 |
| Not infected | 5 (41.7) | 13 (26.5) | 0.3 (0.1-1.5) | 0.144 |
| Condom used, n (%) |  |  |  |  |
| Consistent used (100%) | 5 (41.7) | 8 (16.3) | 1 |  |
| Inconsistent used | 7 (58.3) | 33 (67.3) | 2.9 (0.73-11.75) | 0.126 |
| Condomless | 0 | 8 (16.3) | 0 | 0 |
| Current alcohol use, n (%), |  |  |  |  |
| Yes | 10 (83.3) | 35 (71.4) | 0.5 (0.1-2.5) | 0.407 |
| No | 2 (16.7) | 14 (28.6) | 1 |  |
| Current smoking, n (%), |  |  |  |  |
| Yes | 5 (41.7) | 14 (28.6) | 0.6 (0.2-2.1) | 0.384 |
| No | 7 (58.3) | 35 (71.4) | 1 |  |

Abbreviations: PrEP, Pre-exposure phophylaxis; MSM, Men who have sex with men; GC, Gonococcal; CT, *Chlamydia trachomatis*
