# Supplementary material for: A pilot program of HIV pre-exposure prophylaxis in Thai youth
Source: PLoS One. 2024 Feb 22;19(2):e0298914. doi: 10.1371/journal.pone.0298914 (PMC10883585; doi:10.1371/journal.pone.0298914)
Supplement: S2 Table — (DOCX) [file pone.0298914.s002.docx]

**S2 Table.** TFV-DP concentrations among participants by gender and adherence at week 12 and 24.

| ^†^**TFV-DP concentrations at week 12 (N = 50)** | | | | | | | | | |
| --- | --- | --- | --- | --- | --- | --- | --- | --- | --- |
| **N** | ***Consistent ≥ 4 pills/week*** | | | ***Consistent ≥ 6 pills/week*** | | | ***Consistent ≥ 7 pills/week*** | | |
|  | Women  11 | Men  39 | Total  50 | Women  11 | Men  39 | Total  50 | Women  11 | Men  39 | Total  50 |
| **Mean TFV-DP fmol/punch (SD)** | 1308.3  (260.1) | 1477.3  (833.9) | 1457.0  (785.6) | 1440.5  (174.7) | 1688.1  (891.7) | 1660.6  (842.5) | 1440.5  (174.7) | 1753.9  (938.1) | 1714.8  (880.9) |
| **Adherence by TFV-DP level,**  **n (%)** | 3  (27.3) | 22  (56.4) | 25  (50.0) | 2  (18.2) | 16  (41.0) | 18  (36.0) | 2  (18.2) | 14  (35.8) | 16  (32.0) |
| **Adherence by self-reported,**  **n (%)** | 9  (81.8) | 37  (94.9) | 46  (92.0) | 7  (63.6) | 27  (69.2) | 34  (68.0) | 7  (63.6) | 18  (46.2) | 25  (50.0) |
| ^†^**TFV-DP concentrations at week 24 (N = 49)** | | | | | | | | | |
| **N** | ***Consistent ≥ 4 weekly dose*** | | | ***Consistent ≥ 6 weekly dose*** | | | ***Consistent ≥ 7 weekly dose*** | | |
|  | Women  11 | Men  38 | Total  49 | Women  11 | Men  38 | Total  49 | Women  11 | Men  38 | Total  49 |
| **Mean TFV-DP fmol/punch.**  **(SD)** | 1293.7  (548.2) | 1474.6  (540.8) | 1446.0  (530.8) | 1527.5  (522.6) | 1654.1  (504.4) | 1636.0  (488.2) | 1897.0  (0.00) | 1742.0  (509.3) | 1756.1  (485.4) |
| **Adherence by TFV-DP level,**  **n, (%)** | 3  (27.3) | 16  (42.1) | 19  (38.8) | 2  (18.2) | 12  (31.6) | 14  (28.6) | 1  (9.1) | 10  (26.3) | 11  (22.5) |
| **Adherence by self-reported,**  **n (%)** | 6  (54.5) | 30  (78.9) | 36  (73.5) | 4  (36.4) | 20  (52.6) | 24  (48.9) | 4  (36.4) | 15  (39.5) | 19  (38.8) |

^†^TFV-DP categories were based on ≥ 700 fmol/punch for ≥ 4 doses/week, ≥ 1050 fmol/punch for ≥ 6 doses/week, and ≥ 1250 fmol/punch for 7 doses/week.
