# Supplementary material for: A pilot program of HIV pre-exposure prophylaxis in Thai youth
Source: PLoS One. 2024 Feb 22;19(2):e0298914. doi: 10.1371/journal.pone.0298914 (PMC10883585; doi:10.1371/journal.pone.0298914)
Supplement: S3 Table — (DOCX) [file pone.0298914.s003.docx]

**S3 Table****.** Factors associated with adherence to pre-exposure prophylaxis treatment among participants retained at week 12.

| **Variables** | **Consistent < 6 pills/week**  **(<1050 fmol/ punch)**  **(N = 32)** | | **Consistent ≥ 6 pills/week**  **(≥1050 fmol/punch)**  **(N=18)** | **P-value** | **Multivariate analysis** | | |
| --- | --- | --- | --- | --- | --- | --- | --- |
|  |  |  |  |  | **Adjusted OR**  **(95% CI)** | | **P-value** |
| Biological sex, n (%) |  | |  |  |  |  | |
| Female | | 9 (28.1) | 2 (11.1) |  | - | - | |
| Male | | 23 (71.9) | 16 (88.9) | 0.178 |  |  | |
| Age at enrolment, median (range), year | | 17.9 (14.9-20.8) | 19.8 (15.7-20.9) |  |  |  | |
| < 18 | | 16 (50.0) | 5 (27.8) |  | - | - | |
| ≥ 18 | | 16 (50.0) | 13 (72.2) | 0.132 |  |  | |
| Enrolment clinic, n (%) | |  |  |  |  |  | |
| Adult HIV clinic | | 1 (3.1) | 3 (16.7) |  |  |  | |
| Private sexual health clinic | | 18 (56.3) | 12 (66.7) | 0.215 | - | - | |
| Paediatric HIV clinic | | 13 (40.6) | 3 (16.7) | 0.052 |  |  | |
| Prior HIV testing; n (%) | |  |  |  |  |  | |
| Yes | | 18 (56.3) | 15 (83.3) | 0.061 | - | - | |
| No | | 14 (43.8) | 3 (16.7) |  |  |  | |
| Risks to take PrEP, n (%) | |  |  |  |  |  | |
| Serodiscordant | |  |  |  |  |  | |
| Yes | | 4 (12.5) | 3 (16.7) | 0.685 | - | - | |
| No | | 28 (87.5) | 15 (83.3) |  |  |  | |
| Inconsistent condom use | |  |  |  |  |  | |
| Yes | | 26 (81.3) | 18 (100.0) | 0 | - | - | |
| No | | 6 (18.8) | 0 |  |  |  | |
| MSM | |  |  |  |  |  | |
| Yes | | 17 (53.1) | 16 (88.9) | **0.018** | **53.2**  **(1.6-1181)** | **0.027** | |
| No | | 15 (46.9) | 2 (11.1) |  |  |  | |
| Having STIs at screening, n (%) | |  |  |  |  |  | |
| Yes | | 3 (9.4) | 9 (50.0) | **0.003** | **85.5**  **(1.9-3816)** | **0.022** | |
| No | | 29 (90.6) | 9 (50.0) |  |  |  | |
| Experienced AEs from PrEP, n (%) | |  |  |  |  |  | |
| Yes | | 5 (15.6) | 1 (5.6) | 0.314 | - | - | |
| No | | 27 (84.4) | 17 (94.4) |  |  |  | |
| Had difficulty taking PrEP*, n (%) | |  |  |  |  |  | |
| Yes | | 20 (62.5) | 3 (16.7) | **0.004** | 0.2  (0.1-1.1) | 0.065 | |
| No | | 12 (37.5) | 15 (83.3) |  |  |  | |
| No. of sex partners in the past month, n (%) | |  |  |  |  |  | |
| ≤ 1 | | 26 (81.3) | 12 (66.7) |  | - | - | |
| ≥ 2 | | 6 (18.8) | 6 (33.3) | 0.252 |  |  | |
| Decreased condom used while taking PrEP, n (%) | |  |  |  |  |  | |
| Yes | | 5 (15.6) | 2 (11.1) | 0.660 | - | - | |
| No | | 27 (84.4) | 16 (88.9) |  |  |  | |
| Current alcohol use, n (%) | |  |  |  |  |  | |
| Yes | | 25 (78.1) | 14 (77.8) | 0.977 | - | - | |
| No | | 7 (21.9) | 4 (22.2) |  |  |  | |
| Current smoking, n (%) | |  |  |  |  |  | |
| Yes | | 10 (31.3) | 4 (22.2) | 0.497 | - | - | |
| No | | 22 (68.8) | 14 (77.8) |  |  |  | |

*Based on stigma, concern others would see pills, social pressures, or a combination of these factors
