# Supplementary material for: A pilot program of HIV pre-exposure prophylaxis in Thai youth
Source: PLoS One. 2024 Feb 22;19(2):e0298914. doi: 10.1371/journal.pone.0298914 (PMC10883585; doi:10.1371/journal.pone.0298914)
Supplement: S4 Table — (DOCX) [file pone.0298914.s004.docx]

**S4 Table****.** Factors associated with adherence to pre-exposure prophylaxis treatment among male participants retained at week 12.

| **Variables** | **Consistent < 4 pills/week**  **(<700 fmol/ punch)**  **(N = 17)** | **Consistent ≥ 4 pills/week**  **(≥ 700 fmol/punch)**  **(N=22)** | **P-value** | **Multivariate analysis** | | | |
| --- | --- | --- | --- | --- | --- | --- | --- |
|  |  |  |  | **Adjusted OR**  **(95% CI)** | | | **P-value** |
| Age at enrolment, median (range), year | 17.9 (15-20.8) | 19.1 (15.6-20.9) |  |  | | |  |
| < 18 | 9 (52.9) | 8 (36.4) |  | - | | | - |
| ≥ 18 | 8 (47.1) | 14 (63.6) | 0.303 |  | | |  |
| Enrolment clinic, n (%) |  |  |  |  | | |  |
| Adult HIV clinic | 10 (58.8) | 4 (18.2) |  |  | | |  |
| Private sexual health clinic | 7 (41.2) | 16 (72.7) | **0.019** | - | | | - |
| Paediatric HIV clinic | 0 | 2 (9.1) |  |  | | |  |
| Prior HIV testing; n (%) |  |  |  |  | | |  |
| Yes | 13 (76.5) | 17 (77.3) | 0.953 | - | | | - |
| No | 4 (23.5) | 5 (22.7) |  |  | | |  |
| Risks to take PrEP, n (%) |  |  |  |  | | |  |
| Serodiscordant |  |  |  |  | | |  |
| Yes | 2 (11.8) | 2 (9.1) | 0.785 | - | | | - |
| No | 15 (88.2) | 20 (90.9) |  |  | | |  |
| Inconsistent condom used |  |  |  |  | | |  |
| Yes | 13 (76.5) | 21 (95.5) | 0.112 | - | | | - |
| No | 4 (23.5) | 1 (4.5) |  |  | | |  |
| MSM |  |  |  |  | | |  |
| Yes | 12 (70.6) | 21 (95.5) | 0.060 | **-** | | | **-** |
| No | 5 (29.4) | 1 (4.5) |  |  |  | | |
| Having STIs at screening, n (%) |  |  |  |  |  | | |
| Yes | 1 (5.9) | 8 (36.4) | **0.049** | **-** | **-** | | |
| No | 16 (94.1) | 14 (63.6) |  |  |  | | |
| Experienced AEs from PrEP, n (%) |  |  |  |  |  | | |
| Yes | 4 (23.5) | 1 (4.5) | 0.112 | - | - | | |
| No | 13 (76.5) | 21 (95.5) |  |  |  | | |
| Had difficulty taking PrEP*, n (%) |  |  |  |  |  | | |
| Yes | 10 (58.8) | 6 (27.3) | 0.052 | - | - | | |
| No | 7 (41.2) | 16 (72.7) |  |  |  | | |
| No. of sex partner in the past month, n (%) |  |  |  |  |  | | |
| ≤ 1 | 14 (82.4) | 14 (63.6) | 0.206 | - | - | | |
| ≥ 2 | 3 (17.6) | 8 (36.4) |  |  |  | | |
| Decreased condom use while taking PrEP, n (%) |  |  |  |  |  | | |
| Yes | 3 (17.6) | 2 (9.1) | 0.435 | - | - | | |
| No | 14 (82.4) | 20 (90.9) |  |  |  | | |
| Condomless sexual intercourse in the past 3 months, n (%) |  |  |  |  |  | | |
| Yes | 8 (47.1) | 18 (81.8) | **0.028** | **6.0**  **(1.0-34.9)** | **0.045** | | |
| No | 9 (52.9) | 4 (18.2) |  |  |  | | |
| Current alcohol use, n (%) |  |  |  |  |  | | |
| Yes | 13 (76.5) | 15 (68.2) | 0.570 | - | | - | |
| No | 4 (23.5) | 7 (31.8) |  |  | |  | |
| Current smoking, n (%) |  |  |  |  | |  | |
| Yes | 5 (29.4) | 4 (18.2) | 0.413 | - | | - | |
| No | 12 (70.6) | 18 (81.8) |  |  | |  | |

*Based on stigma, concern others would see pills, social pressures, or a combination of these factors
