# Supplementary material for: A pilot program of HIV pre-exposure prophylaxis in Thai youth
Source: PLoS One. 2024 Feb 22;19(2):e0298914. doi: 10.1371/journal.pone.0298914 (PMC10883585; doi:10.1371/journal.pone.0298914)
Supplement: S5 Table — (DOCX) [file pone.0298914.s005.docx]

**S5 Table.** Factors associated with adherence to pre-exposure prophylaxis treatment among male participant retained at week 24.

| **Variables** | **Consistent < 4 pills/week**  **(<700 fmol/ punch)**  **(N = 22)** | **Consistent ≥ 4 pills/week**  **(≥ 700 fmol/punch)**  **(N=16)** | **P-value** | **Multivariate analysis** | | |
| --- | --- | --- | --- | --- | --- | --- |
|  |  |  |  | **Adjusted OR**  **(95% CI)** | | **P-value** |
| Age at enrolment, median (range), year | 17.2 (15-20.8) | 19.5 (15.7-17.9) |  |  | |  |
| < 18 | 13 (59.1) | 4 (25.0) |  | - | | - |
| ≥ 18 | 9 (40.9) | 12 (75.0) | **0.042** |  | |  |
| Enrolment clinic, n (%) |  |  |  |  | |  |
| Adult HIV clinic | 12 (54.5) | 2 (12.5) |  |  | |  |
| Private sexual health clinic | 10 (45.5) | 13 (81.3) | **0.018** | **7.8**  **(1.4-43.1)** | | **0.018** |
| Paediatric HIV clinic | 0 | 1 (6.3) |  |  | |  |
| Prior HIV testing, n (%) |  |  |  |  | |  |
| Yes | 15 (68.2) | 14 (87.5) | 0.180 | - | | - |
| No | 7 (31.8) | 2 (12.5) |  |  | |  |
| Risks to take PrEP, n (%) |  |  |  |  | |  |
| Serodiscordant |  |  |  |  | |  |
| Yes | 2 (9.1) | 1 (6.3) | 0.750 | - | | - |
| No | 20 (90.9) | 15 (93.8) |  |  | |  |
| Inconsistent condom use |  |  |  |  | |  |
| Yes | 17 (77.3) | 16 (100.0) | - | - | | - |
| No | 5 (22.7) | 0 |  |  | |  |
| MSM |  |  |  |  | |  |
| Yes | 16 (72.7) | 16 (100.0) | - | **-** | | **-** |
| No | 6 (27.3) | 0 |  |  |  | |
| Having STIs at screening, n (%) |  |  |  |  |  | |
| Yes | 3 (13.6) | 6 (37.5) | 0.098 | **-** | **-** | |
| No | 19 (86.4) | 10 (62.5) |  |  |  | |
| Experienced AEs from PrEP, n (%) |  |  |  |  |  | |
| Yes | 5 (22.7) | 2 (12.5) | 0.428 | - | - | |
| No | 17 (77.3) | 14 (87.5) |  |  |  | |
| Had difficulty taking PrEP*, n (%) |  |  |  |  |  | |
| Yes | 10 (45.5) | 3 (18.8) | 0.096 | - | - | |
| No | 12 (54.5) | 13 (81.3) |  |  |  | |
| No. of sex partner in the past month, n (%) |  |  |  |  |  | |
| ≤ 1 | 17 (77.3) | 9 (56.3) |  | - | - | |
| ≥ 2 | 5 (22.7) | 7 (43.8) | 0.174 |  |  | |
| Decreased condom use while taking PrEP, n (%) |  |  |  |  |  | |
| Yes | 4 (18.2) | 7 (43.8) | 0.094 | - | - | |
| No | 18 (81.8) | 9 (56.3) |  |  |  | |
| Condomless sexual intercourse in the past 3 months, n (%) |  |  |  |  |  | |
| Yes | 11 (50.0) | 11 (68.8) | 0.251 | - | - | |
| No | 11 (50.0) | 5 (31.30) |  |  |  | |
| Current alcohol use, n (%) |  |  |  |  |  | |
| Yes | 17 (77.3) | 12 (75.0) | 0.871 | - | - | |
| No | 5 (22.7) | 4 (25.0) |  |  |  | |
| Current smoking, n (%) |  |  |  |  |  | |
| Yes | 5 (22.7) | 4 (25.0) | 0.871 | - | - | |
| No | 17 (77.3) | 12 (75.0) |  |  |  | |

*Based on stigma, concern others would see pills, social pressures, or a combination of these factors
