# Supplementary material for: A pilot program of HIV pre-exposure prophylaxis in Thai youth
Source: PLoS One. 2024 Feb 22;19(2):e0298914. doi: 10.1371/journal.pone.0298914 (PMC10883585; doi:10.1371/journal.pone.0298914)
Supplement: S6 Table — (DOCX) [file pone.0298914.s006.docx]

**S6 Table.** Self-reported adverse events in adolescents while taking tenofovir disoproxil fumarate-emtricitabine for pre-exposure prophylaxis.

| **Adverse events (N=51)** | **n (%)** |
| --- | --- |
| Appetite loss | 4 (7.8) |
| Nausea | 11 (21.6) |
| Flu-like symptoms | 3 (5.9) |
| Dizziness | 11 (21.6) |
| Vomiting | 2 (3.9) |
| Myalgia | 1 (2.0) |
| Abdominal pain | 1 (2.0) |
| Rashes | 3 (5.9) |
| Not feeling well | 2 (3.9) |
| Diarrhoea | 1 (2.0) |
| Headache | 2 (3.9) |
