# Supplementary material for: A pilot program of HIV pre-exposure prophylaxis in Thai youth
Source: PLoS One. 2024 Feb 22;19(2):e0298914. doi: 10.1371/journal.pone.0298914 (PMC10883585; doi:10.1371/journal.pone.0298914)
Supplement: S7 Table — (DOCX) [file pone.0298914.s007.docx]

**S7 Table.** Bone mineral density and kidney function in adolescents taking tenofovir disoproxil fumarate-emtricitabine for pre-exposure prophylaxis.

| **Bone mineral density (BMD)** | | | |  |
| --- | --- | --- | --- | --- |
| **Variables** | **Baseline (N = 49)** | **Week 24 (N = 49)** | ***P*-value*** |  |
| Z-score, median (range) | 0.3  (-2.1 – 5.1) | 0.3  (-2.4 – 5.2) | 0.90 |  |
| BMD (g/cm^2^), median (range) | 1.10  (0.919 – 1.555) | 1.12  (0.916 – 1.572) | 0.01 |  |
| **Renal function** | | | | |
| **Variables** | **Baseline (N = 50)** | **Week 12 (N = 50)** | ***P*-value*** |  |
| CrCl^†^ mL/min,  median (range) | 114.0  (74.3-217.2) | 116.5  (75.1-211.9) | 0.81 |  |
|  | **Baseline (N = 49)** | **Week 24 (N = 49)** | ***P*-value*** |  |
|  | 115.2  (74.3-217.2) | 122.8  (71.4-220.8) | 0.04 |  |
|  | **Week 12 (N = 49)** | **Week 24 (N = 49)** | ***P*-value*** |  |
|  | 116.6  (75.1-211.9) | 122.8  (71.4-220.7) | 0.07 |  |

*Compared dependent samples by Wilcoxon signed-rank test. †CrCl calculated by Cockcroft-Gault Equation.
